# Plant extractivism in light of game theory: a case study in northeastern Brazil

**DOI:** 10.1186/1746-4269-11-6

**Published:** 2015-02-23

**Authors:** Rafael RV Silva, Laura Jane Gomes, Ulysses Paulino Albuquerque

**Affiliations:** Centro de Ciências Agrárias, Área de Engenharia Florestal, Universidade Federal de Alagoas, BR-104 Norte, Km 85, Mata do Rolo, CEP: 57100-000 Rio Largo, Alagoas, Brazil; Departamento de Ciências Florestais, Universidade Federal de Sergipe, Avenida Marechal Rondon, Jardim Rosa Elze, CEP: 49100-000 São Cristóvão, Sergipe Brazil; Departamento de Biologia, Laboratório de Etnobiologia Aplicada e Teórica, Universidade Federal Rural de Pernambuco, Avenida Dom Manoel de Medeiros, Dois irmãos, CEP: 52171-900 Recife, Pernambuco, Brazil

**Keywords:** Common resources, Human ecology, Conservation, Human adaptative strategies, Forest extractivism

## Abstract

**Background:**

Game theory enables the predictive study of the behavior of agents that recognize the mutual interdependence of their decisions and act rationally and strategically to maximize their own gains. In this paper, the extractivism of pequi (*Caryocar coriaceum* Wittm.) and fava d’anta (*Dimorphandra gardneriana* Tul.) in an area of common use of the semiarid region of northeastern Brazil is described as a model to illustrate a practical application of game theory in the interpretation of the phenomena analyzed in ethnobiological research.

**Methods:**

Field research was conducted in the Araripe-Apodi National Forest. Semi-structured interviews were conducted with 55 informants recognized as knowledgeable and experienced in the extractivism of pequi and fava d’anta in the region. In the interviews, information that could contribute to the identification and understanding of the focal points of extractivism in the region was surveyed. Data were analyzed under an analytical/descriptive approach using the “content analysis” technique.

**Results:**

There was a logic of competitive entrepreneurship around the commercial extractivism of pequi and fava d’anta in the region. Among the extractivists of pequi, one of the main collection rules refers to the prohibition of removing immature fruits by using sticks or shaking the branches. In the extractivism of fava d’anta, no specific rules have been established by collectors, but there is a predominant behavior of withdrawing all available fruits (pods) on a tree in a single visit. In an analysis guided by game theory, the collection decisions adopted by extractivists to maximize gain from the activity can be considered justifiable from the standpoint of economic rationality.

**Conclusions:**

The “game of extractivism” of pequi and fava d’anta operates under conditions similar to the “tragedy of the commons.” In this game, the non-cooperative solution is converted to the Nash equilibrium. The approach used in this study contributed to the identification of strategies and solutions to problems arising from the extractivism of pequi and fava d’anta.

## Resumo

**Contexto:**

A Teoria dos Jogos permite o estudo preditivo do comportamento de agentes que reconhecem a interdependência mútua de suas decisões e agem de forma racional e estratégica, isto é, com interesse em tomar decisões ótimas para aumentar os seus próprios ganhos. Neste artigo, o extrativismo de pequi (*Caryocar coriaceum* Wittm.) e a fava d’anta (*Dimorphandra gardneriana* Tul.), em uma área de uso comum na região semiárida do nordeste do Brasil, é descrito como um modelo para exemplificar uma aplicação prática da teoria dos jogos na interpretação dos fenômenos analisados em uma pesquisa etnobiológica.

**Métodos:**

A pesquisa de campo foi realizada na Floresta Nacional Araripe-Apodi. Entrevistas semi-estruturadas foram realizadas com 55 informantes, reconhecidos como experientes conhecedores do extrativismo do pequi e fava d’anta na região. Nas entrevistas, foram levantadas informações que pudessem contribuir para a identificação e o entendimento dos *pontos focais* do jogo do extrativismo na região. Os dados foram analisados por meio de uma abordagem analítica / descritiva, utilizando a técnica de “análise de conteúdo”.

**Resultados:**

Verificou-se uma lógica de empreendedorismo competitivo em torno do extrativismo comercial de pequi e fava d’anta na região. Entre os extrativistas do pequi, uma das principais regras de coleta refere-se à proibição de remoção de frutos imaturos usando varas ou sacudindo os ramos. No extrativismo da fava d’anta não há regras específicas estabelecidas pelos extrativistas, mas há há um predomínio da retirada todos os frutos (vagens) disponíveis na árvore em uma única visita. Em uma análise guiada pela teoria dos jogos, considera-se que as decisões de coleta adotadas pelos extrativistas, a fim de obter um maior ganho da atividade, são justificáveis do ponto de vista da racionalidade econômica.

**Conclusões:**

O “jogo do extrativismo” de pequi e fava d’anta opera em condições semelhantes à Tragédia dos Comuns. Neste jogo, a solução não-cooperativa se converte no equilíbrio de Nash. A abordagem utilizada neste estudo contribuiu para a identificação de estratégias e soluções para os problemas decorrentes do extrativismo do pequi e fava d’anta.

## Background

Game theory consists of a theoretical and methodological framework that allows the study of the behavior of agents (individuals or groups of individuals) who recognize the mutual interdependence of their decisions and act rationally and strategically, i.e., with an interest towards making optimal decisions to increase their own gains [1, 2]. Through different theoretical models, game theory enables a determination of the necessary conditions for producing the expected results. Overall, this analytical perspective emphasizes the point of view of economic rationality - an approach sometimes criticized in studies guided by other economic aspects influenced by the realization that rationality is not limited to a question of maximization of utility but rather depends on the limitations of information, standards and evolving mental abilities [3].

In recent applications, game theory has gained greater popularity in use for evolutionary biology-related studies [4]. In this vast field, there is a special interest in explaining the behaviors considered more or less fit [1]. Although many combinations of genes can cause certain behavioral tendencies, which share their origin in environmental factors at a level that prevents the determination of genetic or environmental influences on them, in the context of evolutionary biology, “gain” is considered to mean reproductive success. Therefore, rational decisions would be related to strategies that increase reproductive success (gain) of the species, and the evolutionary process of natural selection would be dominated by rational decisions [4].

We consider evidence in the field of evolutionary biology to serve as important *insights* for the application of game theory in the field of ethnobiology. In ethnobiological research, we seek to understand the theory of evolution not just in its biological context because rules, codes of conduct, lifestyles, beliefs, and other human cultural traits also result in and influence evolutionary processes. In this context, an important aspect in ethnobiological studies on the use of plant resources should be the investigation of competition or cooperation existence, both in resource collection and marketing used by human populations.

The experiences presented in the scientific literature allow the consideration that although local populations often have a rich knowledge of the ecosystems with which they interact [5, 6], which may be linked to their cultural identity [7], this does not ensure that they will develop the same practices that contribute to the conservation of the resources used [8, 9]. According to Belcher and Kusters [10], in situations where the extractivists need to meet their immediate needs for sustenance, they do not always consider the risks of overexploitation. Therefore, the following questions arise: in regard to the search for resources, can game theory help explain which behaviors have a greater or lesser prevalence in a given culture? What are the practical implications of this understanding in the search for models of management of natural resources for human populations and users of common resources?

In the scientific literature, the most tested and discussed idea for issues of this nature is known as “the tragedy of the commons” as coined by Hardin [11]. According to this author, when a natural resource is in common use, there is a tendency for competition to lead to exhaustion. This idea was postulated on an alleged situation that occurred in Europe in the Middle Ages, where shepherds could freely feed their sheep on lands without a specific owner. In this case, the most advantageous outcome for every shepherd would always be to increase the flock. Assuming everyone acted in this manner, at some point the common pasture would be overexploited and soon exhausted. Consequently, there would be a lack of resources for all animals, thus leading to a tragedy of the commons. In this game, the conflict is the decision whether to extensively use a common resource with an understanding that extensive use will lead to its exhaustion, causing damage to everyone involved.

The situation described in the classic format of the tragedy of the commons is a non-cooperative game. In this game, the best solution for the players would be achieved through mutual cooperation, management mechanisms and access regulation to the common resource. However, by the logic of economic rationality, the uncooperative decision (maximizing the flock) represents the best individual result because the best response to competitors increasing their flock is to do the same. Thus, the solution in which all players increase their flock represents a “Nash equilibrium” [12], which is the best response in relation to the rational choices of the other players. It appears that in this game of the commons, the theoretical solution does not correspond to the best collective result.

Many of the problems related to common resources management involve situations in which the rational behavior for the self-interest of individuals leads to socially suboptimal outcomes. In this article, we discuss these problems in the context of extractivism of two important species of Brazilian sociobiodiversity, pequi (*Caryocar coriaceum* Wittm.) and fava d’anta (*Dimorphandra gardneriana* Tul.), in an area of common use in the semiarid region of northeastern Brazil. We use this case study as a model to illustrate a practical application of game theory in the interpretation of the phenomena analyzed in ethnobiological research. In this regard, we assume that the case study will present an analogous situation to the “tragedy of the commons” and that extractivists will adopt specific mechanisms for managing these resources in order to prevent its exhaustion. We evaluated this idea in a descriptive way, considering both the relations among extractivists and among extractivists and the formal institutions involved in the activities.

## Methods

### Study area

**Figure 1:**
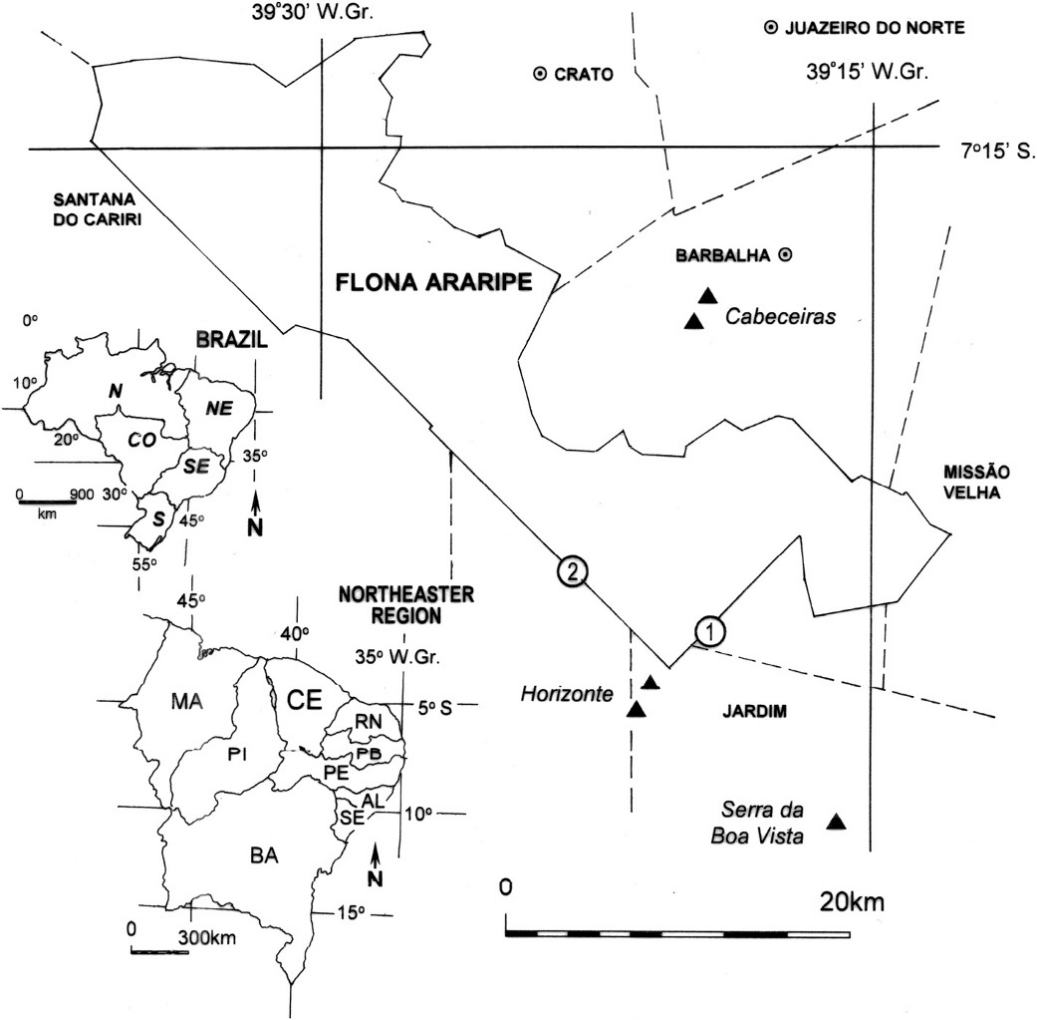
**Location of the study area in the FLONA of Araripe, CE, northeastern Brazil, where ▲ = Point of purchase and drying of fava d’anta; ① = “Barreiro Novo” camp of extractivists of Pequi; and ② “Siriqueira” camp of extractivists of Pequi.**

Field research was conducted in the Araripe-Apodi National Forest (FLONA) (Figure 1), which is classified by Brazilian law as a nature conservation unit (NCU) under the grouping of sustainable use, for which the basic objective is to match nature conservation with the sustainable use of its natural resources [13]. In this region, composed of Cerrado vegetation (Brazilian Savanna) inserted in the semiarid region of northeastern Brazil, the Horizonte community (S 07° 29′ 36.9″; W 39° 22′ 02.6″) (Figure 1) was identified as the community most dependent on the sale of forest products obtained by plant extractivism. Much of the marketed product is obtained in the FLONA, comprising an area of common use by the extractivist community.

Approximately 1,120 people reside in the Horizonte community, but the number of residents varies throughout the year; due to the limited employment opportunities available in the region, it is common for people to migrate to other regions in search of work during some months of the year. During the period in which the survey was conducted, there were approximately 462 people over 18 years old in the community according to the information reported by residents and provided by local health workers. Out of this population, it was estimated that approximately 282 individuals were practitioners of plant extractivism for commercial purposes. Among these, the extractivism of pequi (*Caryocar coriaceum* Wittm., Caryocaraceae) and fava d’anta (*Dimorphandra gardneriana* Tul., Fabaceae) stood out as the most economically important productive activities to the local people.

The fruits of *C. coriaceum* are traded in the FLONA region with food for medicinal purposes both *in natura* and processed in the form of oil, which is part of the local medical, popular and regional culinary traditions. The economic importance of *D. gardneriana* is due to the high content of the flavonoids rutin and quercetin in its pods; both are widely used by pharmaceutical industries, which are responsible for the commercial demand for fava d’anta in the region. Production chains formed from the extractivism of these two species, their socioeconomic aspects, the dynamics of production and marketing in the Horizonte community are treated in greater detail in another publication by the same authors.

### Sampling and data analysis

Semi-structured interviews were conducted with 55 informants, among them extractivists, processors, middlemen and others recognized as knowledgeable and experienced in the extractivism of pequi (*C. coriaceum*) and fava d’anta (*D. gardneriana*) in the region. These informants, considered local experts, were selected through nonprobabilistic sampling by means of an adaptation of the “snowball” technique [14], in which one informant was interviewed and requested at the end to indicate other potential informants, and so on, until the point when the indicated informants or information obtained began to repeat, indicating field exhaustion. The interviews were conducted in community headquarters such as in extractivist camps and purchase points associated with activities (Figure 1).

In the interviews, information that could contribute to the identification and understanding of the focal points of the extractivism game in the region was surveyed as a way of discussing the specific conditions in which the extractivists, in strategic interaction, tend to adopt certain behaviors in order to obtain greater success in collecting or greater economic return for the activity. In addition, direct observations of extractivist activities were conducted with the presence of the researcher in the community during harvest periods.

Use of the information provided was approved by the research participants who each signed an informed consent form in compliance with the requirements of Brazilian legislation (Resolution No. 466/2012 of the National Board of Health). Data were analyzed under an analytical/descriptive approach using the technique of “content analysis” [15], in which the data were organized, the content of responses were identified, and inferences were drawn regarding to the theoretical discussion proposed by the study.

## Results and discussion

In general, extractivists have poor socioeconomic conditions with few job options for income generation. This context weakens the community, which faces difficulties in organizing production in a cooperative manner to ensure greater economic benefits and effective control of the activity. Thus, there was a logic of competitive entrepreneurship around the commercial extractivism of pequi (*C. coriaceum*) and fava d’anta (*D. gardneriana*.) in the region.

One piece of evidence in this sense refers to the history of projects and actions aimed at promoting the production and marketing of pequi cooperatively in the community through the implementation of a processing unit for oil production installed by the Associação de Moradores de Horizonte. This action was promoted by an NGO with the support of the UCN management body among other funders. However, the results were unsatisfactory because the extractivists involved considered it more advantageous to produce independently because they could negotiate prices different from the marketing values prefixed by the association.

In the case of fava d’anta, a similar pattern was evident, but it was focused more on the suppliers, i.e., intermediaries hired by the consumer company to organize production. Although the managing agency of the FLONA requires the marketing of pods through neighborhood associations, the “supplier” (intermediate) continues to play a strong role in the production process. Thus, the requirement does not fully meet the objective of ensuring greater community control of the activity, a fact aggravated because the few suppliers operating in the region (only four), considering independent action to be more advantageous, do not express an interest in consolidating an extractivist cooperative. In this context, during the period of this survey, the purchasing company also did not develop any action to ensure a greater bond with or commitment to the extractivist community, such as long-term contracts.

Among the pequi extractivists, one of the main collection rules refers to the prohibition of removing immature fruits by using sticks or shaking the branches. This practice, generally motivated by an interest for individual maximization of earnings, is repudiated by most extractivists under the following allegations:

1. it affects the final consumer by generating a product of inferior quality to the fruit collected on the ground;
2. it is damaging to other extractivists; and
3. it harms the tree and negatively affects the production in later years. In the extractivism of fava d’anta, there are no specific rules established by collectors, but there was repudiation for felling these trees or breaking branches when collecting. Among the collection practices, there is a predominance of withdrawing all the fruits (pods) available on the tree in a single visit.

In an analysis guided by game theory, one can consider the collection decisions adopted by extractivists maximize individual gain from the activity to be justifiable from the standpoint of economic rationality. The decision to harvest immature fruits, as occurs with pequi, or collect all the fruits available in the tree, as with fava d’anta, has a simple explanation: in a context of competition searching for the resource, the prevailing thought is, “*If I do not do it, someone else will do it*.” [16] However, this choice, when adopted by all extractivists, tends to increase harvesting pressure, thus increasing the risk of resource depletion.

**Figure 2:**
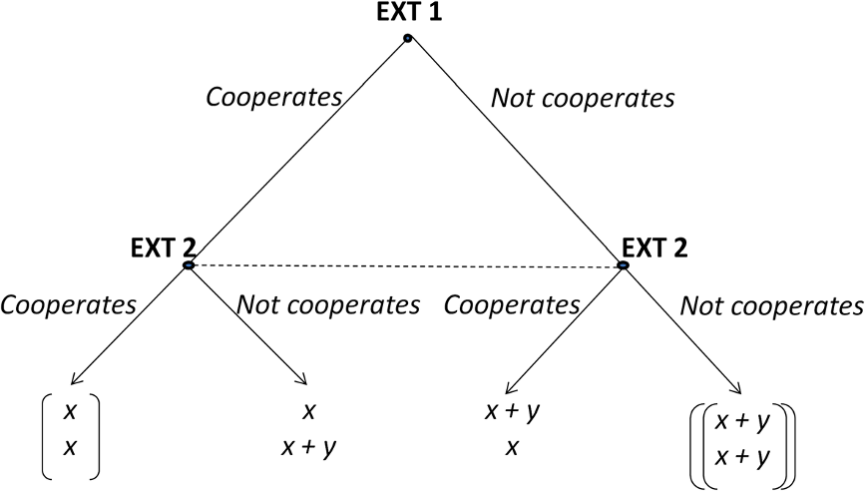
**Adaptation of prisoners’ dilemma as a “game of extractivism” in the decision tree scheme, in which EXT1 = Extractivist 1 and EXT2 = Extractivist 2.** Note: Outcomes represented in the top row of the pairs presented at the end of the tree are the extractivist 1; the result highlighted in brackets is the cooperative solution, and the non-cooperative solution is between the double brackets (the fall of immature pequi fruits or the collection of all the pods of fava d’anta aimed at increasing the individual gains).

Therefore, one can consider that the conditions of the “game of extractivism” are sufficient for rational agents to convert to non-cooperation in the Nash equilibrium, i.e., the best possible response when considering the decision that other players should take. In this “game,” the pequi fruits collected from the ground represent the “cooperative” gain, and the fruits already fallen to the ground added to the downed fruits represent the uncooperative gain (maximizing individual return). In the case of fava d’anta, one can employ the same logic, but the non-cooperative gain would be represented by the removal of all pods at collection. In Figure 2, the “game of extractivism” is represented in a decision tree scheme.

The “game of extractivism” involves numerous players and occurs in “successive rounds,” making it a social dilemma that takes the theoretical dimensions of the tragedy of the commons game. Based on game theory assumptions, the cooperative solution (represented in single brackets in Figure 2) can be considered to be more advantageous in the case of sharing rules for collecting and ensuring continuity of the game, hence ensuring greater chances of the perpetuation of extractivist activity and resource conservation. The non-cooperative solution (represented in double brackets in Figure 2), which is a Nash equilibrium in the described context, would always represent the best solution whenever the perspective of continuing the game was not in play and there were no commitments previously established among players; however, it leads to greater pressure on the resource, thereby increasing the chances of activity decline and, consequently, a “tragedy of the commons.”

The extractivism of pequi usually occurs under a logic of “successive rounds” because the same tree is collected over the same crop and the activity will take place over an indefinite number of crops. Thus, the extractivism of pequi presents, from the standpoint of economic rationality applied to game theory, the minimum conditions necessary for cooperation among players (extractivists). However, during the final round (e.g., last collection or late harvest), when the game inevitably occurs in a “single round”, the rational solution happens to be non-cooperation.

In the extractivism of fava d’anta, the conditions observed allow for consideration under an analytical perspective guided by game theory. The activity occurs in a logic of a single round, as it is usually collected only once per tree, and there are no guaranteed commitments for purchases in later harvests or legal mechanisms to ensure a bond between the company, suppliers and extractivists for purchase in subsequent years. These extractivists are only informed about the company’s interest and the quantity to be ordered in the period before the harvest. Therefore, they do not have any information about future demands - which complicates the implementation of strategies including community management of the species. In this case, the cooperative solution would only represent a benefit to the extractivists of fava d’anta by establishing long-term contracts, which guarantee the continuity of the game through successive rounds as well as a better distribution of the benefits from the activity.

Mechanisms that promote cooperation among extractivists should be prepared in the “game of extractivism.” According to Ostrom [17], the management of common goods for long periods can be ensured through the establishment of institutional arrangements generally composed of several elements. In the case of extractivism of pequi and fava d’anta, this would entail the strengthening of associations, negotiation of grants and policies regarding minimum prices, agreements, rules, long-term contracts, monitoring and conflict resolution.

In the study area, the regulations imposed by the managing agency (formal rules) and the rules passed down orally by the owners of collection sites or among extractivists (informal rules) have apparently contributed to the control of extractivism. According to Ostrom et al. [18], submitting extractivism to a set of formal and informal rules can have a positive effect for greater control of the collection. These authors purport that overexploitation in common areas can be avoided when users have regulatory mechanisms for collection.

One such case was observed by Yang et al. [19] among extractivists of “matsutake” mushroom in northwest Yunnan Province, China, who developed rotating collection systems, collective management, labor savings and benefit sharing that reduced the harvest pressure on the resource to solve the problem of unequal distribution of resources. Experiences like this serve as evidence that extractivist activity becomes a viable strategy for conservation of the species when there is organization and cooperation among the social actors involved.

The repudiation of extractivists from Horizonte/CE for the practice of felling immature pequi fruits and the attacks to trees serve as examples of local regulatory mechanisms of activity. This reinforces our vision that in a situation analogous to the “tragedy of the commons,” foragers would draw up arrangements for the management of resources to prevent the exhaustion of these resources. However, these control rules could not completely eliminate the assumption of inappropriate collection practices in the region. In a study conducted in the same community, Souza-Júnior et al. [20] found that 19.5% of extractivist respondents claimed to have collected immature pequi fruits when ripe fruits were not found on the ground.

It is worth noting that the land status of the main collecting areas in the study area, set in a conservation of sustainable use area, contributes to the greater control of activities. The managing agency plays an important role in regulating and, even if precariously, supervising the activity. Furthermore, actions that would promote the production of seedlings and stimulate enrichment plantation in conservation units by the authorizing agency may have positive effects on the extractivist economy over the long term. Such actions, which constitute the management of natural populations, may help to ensure that resources remain available and accessible to extractivism in the future.

## Conclusion

The “game of extractivism” of pequi (*Caryocar coriaceum* Wittm.) and fava d’anta (*Dimorphandra gardneriana* Tul.) operates under conditions similar to the tragedy of the commons. In this game, the non-cooperative solution is converted to the Nash equilibrium whenever there are no guaranteed commitments or consideration of future rounds. The search for the maximization of individual gains is justified in the context studied, where there are few job options and strong competition in the search for extractivist resources in an area of common use. The implications of this scenario, as with the predictions of game theory, tend to generate negative outcomes for community.

In this regard, the case study revealed that those involved in extractive activities have developed specific mechanisms for the management of resources to prevent its exhaustion. Although this fact corroborated with our idea, it does not allow us to infer that the extractive activity will be insured against the risk of a tragedy of the commons. Thus, we conclude that “game of extractivism” of pequi (*Caryocar coriaceum* Wittm.) and fava d’anta (*Dimorphandra gardneriana* Tul.) requires the implementation of mechanisms capable of making advantageous collective action and cooperation among stakeholders. Based on the information generated, it becomes possible to identify alternative strategies to the problems arising from this game, which may be important for resource management in common use.

The approach proposed in this study applies the concepts of game theory to ethnobiology. This initial experience has proven to be a practical example of the contribution of game theory to ethnobiological surveys on extractivist populations. Larger, ongoing efforts are necessary to deepen this understanding in the field of ethnobiology.
